# Female Reproductive Factors and Risk of Mild Cognitive Impairment and Dementia: The HUNT Study

**DOI:** 10.14283/jpad.2024.46

**Published:** 2024-03-05

**Authors:** Yehani Wedatilake, C. Myrstad, S. E. Tom, B. H. Strand, S. Bergh, G. Selbæk

**Affiliations:** 1 Norwegian National Centre for Ageing and Health, Vestfold Hospital Trust, Tønsberg, Norway; 2 Research Centre for Age-related Functional Decline and Diseases, Innlandet Hospital Trust, Ottestad, Norway; 3 Department of Geriatric Medicine, Oslo University Hospital, Oslo, Norway; 4 Department of Medicine, Levanger Hospital, Nord-Trøndelag Hospital Trust, Levanger, Norway; 5 Department of Neurology, Sergievsky Center, Vagelos College of Physicians and Surgeons, Columbia University Irving Medical Center, New York, New York USA; 6 Department of Epidemiology, Mailman School of Public Health, Columbia University Irving Medical Center, New York, New York USA; 7 Division for Mental and Physical Health, Norwegian Institute of Public Health, Oslo, Norway; 8 Faculty of Medicine, University of Oslo, Oslo, Norway

**Keywords:** Dementia, Alzheimer’s disease, menopause, menarche, reproductive

## Abstract

**Background:**

More women are living with dementia than men worldwide and there is a need to investigate causes for this female preponderance. While reproductive factors have been investigated as risk factors, the results are conflicting. We aim to clarify this using a large cohort with a long observation time, adjusting for multiple health and lifestyle variables and encompassing a wider range of cognitive impairment.

**Objective:**

To study the association between menopause age, menarche age and risk of and risk of mild cognitive impairment (MCI) and dementia.

**Setting:**

The Trøndelag Health study (HUNT), a longitudinal population health study in Norway (1984–2019).

**Participants:**

Women who were ≥70 years in 2017–2019 were assessed for cognitive impairment.

**Measurements:**

Data on menopause age and menarche age were obtained from questionnaires. Diagnosis of MCI or dementia was set using a standardised procedure by a diagnostic group of nine physicians. Multinomial logistic regression was used to study the association between menopause age, menarche age and risk of MCI and dementia with adjustment for birth year, education, smoking, ApoE4, number of children, diabetes, body mass index, alcohol use and physical inactivity.

**Results:**

We evaluated 5314 women where 900 (16.9%) had dementia, and 1747 (32.8%) had MCI. Multiple adjusted relative risk ratio (RRR) and 95% confidence intervals (CI) for dementia were: 0.96(95%CI 0.95–0.98) (p<0.001) for menopause age, 0.97(95%CI 0.94–0.99) (p=0.007) for natural menopause age (excluding hysterectomy and/or oophorectomy<55 years) and 0.97(95%CI 0.95–0.99) (p<0.001) for reproductive span (menopause age minus menarche age). Menopause age <45years was associated with a 56% higher risk compared to mean menopause age 50 years. We found no significant associations between menarche age and dementia and no associations with MCI.

**Conclusions:**

Older menopause age and longer reproductive span corresponding to longer oestrogen exposure were associated with a lower dementia risk. Future studies should explore therapeutical options to offset this risk in women.

**Electronic Supplementary Material:**

Supplementary material is available in the online version of this article at 10.14283/jpad.2024.46.

## Introduction

**T**here are more women than men living with dementia worldwide with some studies reporting that as many as two-thirds of people with Alzheimer’s dementia are women (1, 2). One explanation which has consistently been given is that women have a longer life expectancy compared to men; advancing age is the most significant risk factor for dementia, and this would mean that more women would live long enough to develop dementia (3). However there is also higher prevalence of dementia among women compared to men even when considering the same age groups (4). One theory is “survival bias” where men who survive to an older age may have a better cardiovascular risk profile which leads to a lower risk for dementia compared to women of the same age (5).

The causes for observed sex-differences are complex and longer life expectancy, reproductive/hormonal factors, environmental factors, social roles/behaviours, and education opportunities are all likely to contribute to varying levels (3).

The data from the Framingham Heart Study demonstrated that the estimated lifetime risk for Alzheimer’s disease related dementia at age 45 years was 20 percent for women and 10 percent for men (5). A meta-analysis of cognitive deterioration in men and women also revealed that women with Alzheimer’s disease had worse cognition across all cognitive domains than men, implying that women may be disproportionately affected by the clinical symptoms of dementia (6).

Therefore, there is a need to investigate the causes which contribute to the predominance of female dementia cases. There is growing evidence that reproductive factors during the female lifetime may affect the lifetime risk of dementia. A history of preeclampsia is associated with higher risk of cognitive decline in later life (7). The Gothenburg cohort study showed that women who experienced a longer reproductive period (years from menarche to menopause) had a higher risk of dementia whereas the Kaiser Permanente Study and the Singapore Chinese Health Study described a greater risk of dementia in women with shorter reproductive span (8–10). A large UK Biobank study found longer reproductive span, and later menopause, were associated with a lower risk of incident dementia (11). Three studies have also demonstrated that women who underwent ‘surgical’ menopause due to bilateral oophorectomy were at higher risk of dementia, than their counterparts with natural menopause (12).

Given that there are more women living with dementia, it is increasingly acknowledged that dementia in women should be a key priority for global women’s health (13). Identifying any female specific dementia risk factors therefore is highly relevant in this context. The main objective of this study was to study the relationship between female specific reproductive factors menopause age, menarche age and reproductive span and the risk of mild cognitive impairment (MCI) and dementia in a well characterised Norwegian population: Our study used data from the full HUNT4 70+ population with a long observation period, used standardised methodology for classification of cognitive impairment, encompassed a wider age range of cognitive impairment and adjusted for multiple health and lifestyle variables to mitigate confounding. MCI has not previously been studied as a separate endpoint and this study aims to also elucidate if there is an association between reproductive factors and MCI. We also analyse the association between reproductive factors and dementia subtypes.

## Methods

The Trøndelag Health Study (HUNT) is a large population-based longitudinal health study in the county of Nord-Trøndelag in Norway. All residents in the county were extended an invitation to be followed up over time in four successive study phases: HUNT1 (1984–1986), HUNT2 (1995–1997), HUNT3 (2006–2008), and HUNT4 (2017–2019). In 2017–2019 during the fourth wave of the HUNT study, all participants who were 70 years or older were invited to participate in the HUNT4 70+ study where the presence of cognitive impairment was assessed. In the HUNT4 70+ study population, 90.3% participated in at least two earlier waves, and 87.3% had participated in all earlier waves (HUNT1–3) (14). Approximately 230,000 people participated in questionnaire data, about 120,000 in clinical measurements in HUNT and it is the largest data collection from a population in Norway (15, 16).

### Dementia diagnosis at HUNT4

During the HUNT4 study in 2017–2019, all participants who were 70 years old or older were invited to participate in the HUNT4 70+ study to establish the presence of MCI or dementia (17). The HUNT4 70+ group was invited to a cognitive assessment protocol consisting of the Montreal Cognitive Assessment (MoCA) scale, the Word List Memory Task (WLMT) from the Consortium to Establish a Registry of Alzheimer’s disease (CERAD), and the Severe Impairment Battery-8 (SIB-8). The MoCA scale is a multidomain cognitive screening instrument which tests memory, visuospatial and executive functions, naming, attention, abstraction, language, and orientation with scores from 0–30; higher scores indicate better cognitive function (18). The WLMT, a 10-word recall test was used for participants scoring ≥22 points on MoCA, to detect those with mild memory difficulties, not detected by the MoCA (19, 20). Recall scores were recorded as an immediate recall score within three attempts (0–30 points) and after 10 min, as a delayed recall score (0–10 points). For nursing home residents, healthcare workers who knew the participants provided information about the participants’ cognitive symptoms. If residents were considered to have moderate to severe dementia, the Severe Impairment Battery-8 (SIB-8) was used for assessment instead of the MoCA (21). Data for each case was assessed by two experts from a diagnostic group of nine physicians with both scientific and clinical expertise in dementia (specialties: geriatrics, neurology, or old-age psychiatry) and diagnoses were made independently. Each case was assessed and categorised as one of the following: no cognitive impairment, mild cognitive impairment, and dementia. Dementia was further subtyped into Alzheimer’s disease (AD), vascular dementia (VaD), Lewy body dementias (LBD), frontotemporal dementia (FTD), mixed dementia, other specified dementia, and unspecified dementia. LBD, FTD, mixed dementia and other specified/unspecified dementia were classified as other dementias for the purpose of this study. All diagnoses were classified according to the DSM-5 criteria (22). Information for this process was obtained from cognitive tests described above, patient history, activities of daily living, neuropsychiatric symptoms, and a structured interview with next of kin. If the diagnosis between the two experts differed and no consensus was reached, a third expert was consulted.

The average age of menopause in this cohort 49.8 years fell in between HUNT1 and 2. Cognitive assessment was performed only in HUNT4 (2017–2019). Data on cognitive status in the previous waves HUNT1-3 are not available and therefore we do not have information on cognitive status around menopause. Persons with cognitive impairment at the time of menopause were therefore not excluded from the study: However, given the survival after a dementia diagnosis (<10 years) it was assumed that with the long follow up time of approximately >20 years between menopause age and HUNT4 that it was unlikely that participants had dementia when they underwent menopause.

### Reproductive factors

The average age for participants at HUNT1 and 2 were 46.7 years and 56.7 years respectively and the average age of menopause in this cohort 49.8 years fell in between HUNT1 and 2 periods. Since HUNT2 is a much larger survey than HUNT1, where more data were collected for each participant, HUNT2 was chosen as baseline for this study.

Menopause and menarche age data were self-reported and available in HUNT2, 3 and 4 questionnaires. Menopause and menarche age were obtained from HUNT2 and where missing, from HUNT3 or HUNT4 questionnaires. The reproductive span was calculated by subtracting menarche age from menopause age. Number of children, hysterectomy, oophorectomy, and age of surgery were self reported in HUNT2 and 3 questionnaires.

### Other covariates

Information on all covariates were obtained either through structured questionnaires (education level, smoking, diabetes, depression) or through health examinations (body mass index) or by genotyping (ApoE). Education level was obtained from HUNT2 questionnaires but where missing, data from HUNT1 and 4 were used. Education level was categorised as compulsory (1–10 years), secondary (11–13 years) and tertiary (>13 years). BMI data from HUNT2 wave were used and where missing were replaced by BMI data from the HUNT1 survey. Data on presence of diabetes were obtained using the HUNT1 and HUNT2 questionnaires. Where diabetes data were missing in HUNT2, data from the HUNT3 and 4 waves were used if participants confirmed that they had never had diabetes. Smoking status from HUNT2 was used and was recorded as previous/current and never smoked. Missing data in HUNT2 was replaced as never smoked, if participants had recorded status as never smoked in any of the other waves. If status was recorded as ex-smoker or current smoker in HUNT 1 or 3, missing data in HUNT2 were imputed accordingly.

### Assessing depression as a possible mediator

Since older menopause age is associated with a lower risk depression (23), and depression is associated with dementia (24), depression was tested as potential mediator between menopause and dementia. Depression was assessed using the Hospital anxiety and depression scale (HADS) score at HUNT2. A HADS score of 8 or above was considered to be abnormal and indicative of depression. The following analyses were carried out: firstly, by adding in depression into the model to assess if it attenuates the association and secondly by excluding individuals with depression and assessing if the association remains.

### Statistical analysis

Data was analysed using Stata 17.0 software. For this study, the following categories of cognitive function were used as the outcome: mild cognitive impairment (includes both amnestic and non-amnestic mild cognitive impairment), dementia (includes all causes of dementia), and no cognitive impairment. Multiple imputation with chained equation was used for missing data for all variables (20 imputed datasets). Multinomial logistic regression was used to estimate unadjusted and adjusted relative risk ratios, significance levels, and confidence intervals between each individual exposure (age of menarche, age of menopause, age at natural menopause and reproductive span), and risk of MCI and dementia. Some of those with MCI will progress to dementia, while others will not. Thus, the outcome is not strictly ordinal and therefore we used multinomial regression instead of ordinal regression. Menarche age and menopause age were analysed both as a continuous variable and as a categorical variable. Menarche age was analysed in the following categories <12, 12–14, >14 years where the three most frequently experienced menarche ages (12–14 years) were used at the reference category. Menopause age was analysed in the following categories <45 years, 45–47 years, 48–49 years, 50 years (mean, used as reference), 51–52 years, 53–54 years and >54 years. Reproductive span was assessed in the following categories: <30 years, 30–33 years, 34–35 years, 36 years (mean, used as reference), 37–38 years, 39–40 years and >40 years. The analysis was performed using two models: a minimally adjusted model for birth year, and a fully adjusted model for birth year, education level, smoking, ApoE4 allele, number of children, diabetes, BMI, alcohol use and physical inactivity. We also studied MCI and dementia risk of those who had oophorectomy and/or hysterectomy <55 years. An exploratory analysis studying the risk of dementia subtypes as outcomes, namely AD, VaD and other dementias vs. no dementia was performed using multinomial logistic regression using reproductive factors where a significant association was found with dementia.

A sensitivity analysis was performed, where the same analysis was run in a dataset without missing data. Death as a competing risk is an important consideration in an older population and the risk of loss to follow-up due to death is higher among the oldest participants. In the current study, data on death and weights to correct for competing risk regarding death were not available. Therefore, a sensitivity analysis was performed with age stratification, using 80 years as the cut-off, to assess if the estimates were larger for the younger participants which could indicate that death was a competing risk.

## Results

In the HUNT4 70+ study, 5420 participants were women; 102 participants were excluded due to insufficient information to set a diagnosis and 4 participants were excluded since the cause of cognitive impairment was due to another cause other than dementia related. The mean age at HUNT2 was 57.7 years (range 48–82, SD 6.7 years), and at HUNT4 79.7 years (range 70–102, SD 6.7 years). Of the remaining 5314 women, when assessed at HUNT4, 900 (16.9%) had dementia, and 1747 (32.8%) had mild cognitive impairment. The mean age of menarche was 13.5 years (SD 1.4 years), and the mean age of menopause was 49.8 years (SD 4.7 years). The mean length of reproductive span (difference between menopause and menarche age) was 36.4 years (SD 4.9 years). Among those who reported childbirth, 342 (6.6%) had one child, 1591 (31%) had two children, 1673 (32.5%) had three children, and 1286 (25%) had four or more children. Of these, 592 (13.5%) women reported hysterectomy or oophorectomy <55 years. Table 1 shows demographic factors and health characteristics by presence of MCI and dementia.

**Table 1:** Sample characteristics of women in HUNT4 70+ study by cognitive status at HUNT4

		**Cognitive status**		
	**No dementia**	**MCI**	**Dementia**	**Total**
Dementia subtypes				
No dementia	2667 (100)	1747 (100)	0.0 (0)	4414 (83.1)
AD	0.0 (0.0)	0.0 (0.0)	527 (58.6)	527 (9.9)
VaD	0.0 (0.0)	0.0 (0.0)	76 (8.4)	76 (1.4)
Other dementia	0 (0.0)	0 (0.0)	297 (33.0)	297 (5.6)
Age at HUNT1 mean (SD) years	44.8 (5.2)	46.3 (6.6)	52.9 (7.4)	46.7 (6.7)
Age at HUNT2 mean (SD) years	55.8 (5.2)	57.3 (6.6)	63.9 (7.4)	57.7 (6.7)
Age at HUNT3 mean (SD) years	66.8 (5.2)	68.3 (6.6)	74.9 (7.4)	68.7 (6.7)
Age at HUNT4 mean (SD) years	77.8 (5.2)	79.3 (6.6)	85.9 (7.4)	79.7 (6.7)
Body mass index mean (SD) kg/m^2^	26.5 (4.1)	26.8 (4.2)	27.4 (4.6)	26.7 (4.2)
Menarche age mean (SD) years	13.4 (1.4)	13.4 (1.4)	13.6 (1.3)	13.5 (1.4)
Menopause age mean (SD) years	50.1 (4.6)	49.7 (4.7)	49.1 (5.1)	49.8 (4.7)
Natural menopause age mean (SD) years	50.8 (3.8)	50.4 (3.9)	49.7 (4.3)	50.5 (4.0)
Surgical menopause age mean (SD) years	44.9 (6.0)	45.1 (5.8)	45.3 (6.7)	45.0 (6.1)
Reproductive span mean (SD) years	36.7 (4.8)	36.3 (4.9)	35.5 (5.2)	36.4 (4.9)
Hysterectomy age mean (SD) years	49.0 (9.1)	48.4 (9.6)	49.6 (11.8)	48.9 (9.8)
Oophorectomy age mean (SD) years	50.4 (11.1)	49.5 (10.9)	50.5 (13.8)	50.1 (11.7)
Hysterectomy or oophorectomy <55years N(%)				
no	2000 (87.8%)	1212 (85.7%)	573 (83.9%)	3785 (86.5%)
yes	279 (12.2%)	203 (14.3%)	110 (16.1%)	592 (13.5%)
Number of children N(%)				
0	123 (4.7%)	82 (4.8%)	55 (6.8%)	260 (5.0%)
1	145 (5.5%)	124 (7.3%)	73 (9.0%)	342 (6.6%)
2	867 (32.9%)	524 (30.7%)	200 (24.8%)	1591 (30.9%)
3	899 (34.1%)	560 (32.8%)	214 (26.5%)	1673 (32.5%)
4 or more	604 (22.9%)	417 (24.4%)	265 (32.8%)	1286 (25.0%)
ApoE4 allele N(%)				
no	1943 (73.2%)	1237 (71.5%)	515 (59.2%)	3695 (70.3%)
yes	711 (26.8%)	492 (28.5%)	355 (40.8%)	1558 (29.7%)
Education level N(%)				
compulsory	1094 (41.5%)	948 (55.2%)	610 (70.4%)	2652 (50.8%)
secondary or tertiary	1545 (58.5%)	770 (44.8%)	257 (29.6%)	2572 (49.2%)
Smoking status N(%)				
never	1322 (51.0%)	794 (47.3%)	483 (56.4%)	2599 (50.7%)
previous or current	1269 (49.0%)	885 (52.7%)	373 (43.6%)	2527 (49.3%)
Diabetes N(%)				
no	2622 (98.9%)	1687 (98.2%)	826 (96.5%)	5135 (98.3%)
yes	28 (1.1%)	31 (1.8%)	30 (3.5%)	89 (1.7%)
Physical inactivity N(%)				
no	1278 (51.9%)	764 (48.4%)	375 (46.4%)	2417 (49.8%)
yes	1186 (48.1%)	815 (51.6%)	433 (53.6%)	2434 (50.2%)
Alcohol use N(%)				
0–14 units	2291 (99.9%)	1446 (99.8%)	726 (100.0%)	4463 (99.9%)
>14 units	3(0.1%)	3 (0.2%)	0 (0.0%)	6 (0.1%)
Depression N(%)				
no	2102 (91.7%)	1259 (87.7%)	591 (85.8%)	3952 (89.5%)
yes	190 (8.3%)	176 (12.3%)	98 (14.2%)	464 (10.5%)

### Age at menarche and risk of MCI or dementia

Age at menarche was not significantly associated with dementia or MCI where the multiple adjusted relative risk ratios (RRR) were 0.99 (95% confidence interval (CI) 0.92–1.05) for dementia, and 0.97 (95% CI 0.93–1.02) for MCI. When the risk of dementia was examined by studying menarche age in three groups (ages <12 years, 12–14 and >14), using age 12–14 years as reference, there were no significant differences in risk between the groups.

### Menopause age and risk of MCI or dementia

Older age of menopause and longer reproductive life span demonstrated an inverse association with dementia risk (Table 2). When menopause age was analysed as a continuous variable, older menopause age was associated with a lower risk of dementia in fully adjusted model RRR 0.96 (95% CI 0.94–0.98) with 4% lower risk for each year of increasing menopause age. Compared to those who underwent menopause at 50 years, early menopause (women who had a menopause before 45 years of age) were found to have a higher risk of dementia, RRR 1.56 (95% CI 1.12–2.27). To examine the risk of dementia and natural menopause, women who had hysterectomy and/or oophorectomy under the age of 55 years were excluded. The remaining participants (total n=4602, dementia n=756) were analysed to study risk of dementia and natural menopause. Older age of natural menopause was associated with a lower risk of dementia in fully adjusted models RRR 0.97 (95% CI 0.94-0.99). Natural menopause <45 years of age was associated with a higher risk of dementia compared to reference category (natural menopause of 50 years) with a RRR of 1.62 (95% CI 1.06–2.46). A longer reproductive span was associated with RRR of 0.97 (95% CI 0.95–0.99) with a 3% lower risk of dementia for each reproductive year. When reproductive span was examined as categorical variable with seven categories using the mean (36 years) as reference no significant difference in risk was found in the other categories. When the risk of MCI was examined as an endpoint, no significant association was found between menopause age, reproductive span (MCI cases n=1747) or natural menopause age (MCI cases n=1507) and MCI (table 2). Those who underwent surgery (oophorectomy/ hysterectomy <55 years) demonstrated a higher risk of dementia RRR 1.49 (95% CI 1.15–1.94) compared to those who did not have surgery.

**Table 2:** The relationship between reproductive risk factors and mild cognitive impairment/dementia in the HUNT4 70+ group

			**Model 1**		**Model 1**		**Model 2**		**Model 2**	
			**MCI**		**Dementia**		**MCI**		**Dementia**	
	**Total No.**	**No. Cases MCI/ dementia**	**RR ratio (95% CI)**	***p*** **value**	**RR ratio (95% CI)**	***p*** **value**	**RR ratio (95% CI)**	***p*** **value**	**RR ratio (95% CI)**	***p*** **value**
Menarche age (years) *	5314	1747/900	0.98 (0.94–1.02)	0.410	0.99 (0.91–1.05)	0.759	0.97 (0.93–1.02)	0.314	0.99 (0.92–1.05)	0.715
Menarche age										
<12 years	432	151/50	1.04 (0.83–1.30)	0.720	0.96 (0.65–1.40)	0.822	1.05 (0.84–1.31)	0.653	0.95 (0.64–1.40)	0.796
12–14 years	3716	1226/534	ref	ref	ref	ref	ref	ref	ref	ref
>14 years	1166	370/216	0.93 (0.80–1.08)	0.345	0.87 (0.71–1.07)	0.196	0.92 (0.79–1.07)	0.287	0.86 (0.70–1.06)	0.178
Menopause age (years) *	5314	1747/900	0.98 (0.97–0.99)	0.029	0.96 (0.94–0.98)	<0.001	0.99 (0.97–1.00)	0.127	0.96 (0.95–0.98)	<0.001
Menopause										
<45 years	585	189/132	1.07 (0.84–1.36)	0.572	1.63 (1.18–2.25)	0.003	1.03 (0.81–1.31)	0.807	1.56 (1.12–2.27)	0.008
45–47 years	657	222/134	1.06 (0.84–1.33)	0.632	1.10 (0.80–1.51)	0.556	1.03 (0.82–1.30)	0.786	1.07 (0.77–1.49)	0.672
48–49 years	770	258/141	0.99 (0.80–1.24)	0.990	1.09 (0.80–1.49)	0.593	0.99 (0.79–1.22)	0.899	1.12 (0.81–1.54)	0.499
50 years	926	314/150	ref	ref	ref	ref	ref	ref	ref	ref
51–52 years	939	312/143	0.94 (0.76–1.15)	0.550	0.91 (0.86–1.23)	0.543	0.95 (0.77–1.16)	0.605	0.92 (0.68–1.24)	0.584
53–54 years	720	220/100	0.81 (0.65–1.01)	0.058	0.79 (0.57–1.10)	0.164	0.83 (0.66–1.03)	0.095	0.82 (0.59–1.15)	0.253
>54 years	717	232/100	0.91 (0.73–1.13)	0.393	0.90 (0.65–1.25)	0.519	0.93 (0.75–1.65)	0.545	0.95 (0.68–1.33)	0.763
Natural menopause (years) *,†	4602	1507/756	0.98 (0.96–0.99)	0.029	0.94 (0.92–0.96)	<0.001	0.99 (0.97–1.00)	0.200	0.97 (0.94–0.99)	0.007
Natural menopause †	_			_		_		_		_
<45 years	295	92/77	1.02 (0.74–1.41)	0.900	1.71 (1.14–2.56)	0.010	0,97 (0.70–1.35)	0.880	1.62 (1.06–2.46)	0.024
45–47 years	512	176/93	1.05 (0.82–1.35)	0.704	1.08 (0.76–1.54)	0.653	1.02 (0.79–1.32)	0.844	1.03 (0.72–1.47)	0.867
48–49 years	663	221/115	0.99 (0.78–1.23)	0.919	1.03 (0.74–1.45)	0.841	0.97 (0.77–1.22)	0.809	1.04 (0.73–1.47)	0.830
50 years	855	283/143	ref	ref	ref	ref	ref	ref	ref	ref
51–52 years	873	280/138	0.92 (0.74–1.14)	0.457	0.94 (0.69–1.28)	0.687	0.93 (0.75–1.15)	0.506	0.94 (0.69–1.30)	0.718
53–54 years	687	212/94	0.81 (0.64–1.02)	0.074	0.81 (0.58–1.13)	0.210	0.83 (0.65–1.04)	0.108	0.82 (0.58–1.16)	0.273
>54 years	695	231/87	0.91 (0.73.1.14)	0.428	0.87 (0.62–1.23)	0.439	0.94 (0.74.1.18)	0.573	0.92 (0.64–1.30)	0.628
Reproductive span (years) *	5314	1747/900	0.99 (0.97–1.00)	0.062	0.96 (0.95–0.98)	<0.001	0.99 (0.98–1.00)	0.234	0.97 (0.95–0.99)	<0.001
Reproductive span										
<30 years	563	187/129	1.02 (0.78–1.33)	0.898	1.37 (0.95–1.98)	0.093	0.99 (0.75–1.29)	0.925	1.31 (0.90–1.91)	0.156
30–33 years	635	219/130	0.96 (0.74–1.24)	0.759	0.92 (0.64–1.32)	0.642	0.92 (0.71–1.20)	0.533	0.86 (0.59–1.29)	0.431
34–35 years	744	225/151	0.78 (0.61–1.00)	0.052	0.94 (0.67–1.33)	0.739	0.78 (0.61–1.00)	0.058	0.95 (0.67–1.36)	0.793
36 years	567	198/101	ref	ref	ref	ref	ref	ref	ref	ref
37–38 years	1,056	351/153	0.83 (0.66–1.04)	0.116	0.73 (0.52–1.03)	0.074	0.84 (0.67–1.06)	0.143	0.76 (0.54–1.07)	0.112
39–40 years	786	254/114	0.81 (0.64–1.03)	0.095	0.79 (0.56–1.12)	0.186	0.83 (0.65–1.06)	0.131	0.78 (0.55–1.12)	0.181
>40 years	963	313/122	0.80 (0.63–1.01)	0.065	0.73 (0.51–1.03)	0.071	0.82 (0.65–1.04)	0.108	0.75 (0.52–1,07)	0.114
Hysterectomy and/or oophorectomy <55 years *										
yes	707	240/146	1.20 (0.98–1.46)	0.072	1.50 (1.16–1.94)	0.002	1.16 (0.96–1.42)	0.129	1.49 (1.15–1.94)	0.003
no	4607	1,507/754	ref	ref	ref	ref	ref	ref	ref	ref

Model 1 was adjusted for birth year. Model 2 was adjusted for birth year, education level, smoking, ApoE4 allele, number of children, diabetes, body mass index, alcohol use, physical inactivity, when analysing menopause age, natural menopause age and reproductive span as risk factors. Model 2 was adjusted for birth year, education level, smoking, diabetes, alcohol use, physical inactivity, body mass index, when studying menarche age as a risk factor. *as a continuous variable. †natural menopause excluding women who reported hysterectomy and/or oophorectomy <55 years.

### Menopause age and risk of dementia subtypes

There was a significant association between older menopause age/longer reproductive span and AD and VaD, with a greater risk reduction for VaD (7 percent vs 3 percent lower risk for AD for each year free from menopause). However, when those who underwent surgery <55 years were excluded and natural menopause age was studied, the association with dementia subtypes was attenuated, especially for VaD (table 3). When those who had surgery <55 years were studied, a higher risk of VaD was found RRR 2.60 (95% CI 1.48–4.58) compared to those who did not undergo oophorectomy/hysterectomy <55 years.

**Table 3:** The relationship between reproductive factors and dementia subtypes in the HUNT4 70+ group

			**Model 1**		**Model 1**		**Model 1**	
			**AD**		**VaD**		**Other dementia**	
	**Total No.**	**No. Cases AD/VaD/ Other dementia**	**RR ratio (95% CI)**	***p*** **value**	**RR ratio (95% CI)**	***p*** **value**	**RR ratio (95% CI)**	***p*** **value**
Menopause age (years)	5314	527/76/297	0.97 (0.95–0.99)	0.005	0.92 (0.88–0.96)	<0.001	0.98 (0.96–1.00)	0.148
Reproductive span	5314	527/76/297	0.97 (0.95–0.99)	0.008	0.92(0.88–0.96)	<0.001	0.98 (0.95–1.00)	0.0183
Natural menopause age (years)	4580	446/56/245	0.97((0.94–0.99)	0.016	0.94 (0.88–1.01)	0.080	0.98 (0.94–1.01)	0.213
Hysterectomy and/or oophorectomy <55 years								
yes	707	77/20/49	1.22(0.99–1.64)	0.221	2.60(1.49–4.49)	0.001	1.38(0.97–1-97)	0.072
no	4607	450/56/248	ref	ref	ref	ref	ref	ref
			**Model 2**		**Model 2**		**Model 2**	
			**AD**		**VaD**		**Other dementia**	
	**Total No.**	**No. Cases AD/VaD/ Other dementia**	**RR ratio (95% CI)**	***p*** **value**	**RR ratio (95% CI)**	***p*** **value**	**RR ratio (95% CI)**	***p*** **value**
Menopause age (years)	5314	527/76/297	0.97 (0.95–0.99)	0.013	0.93 (0.89–0.97)	<0.001	0.98 (0.96–1.01)	0.210
Reproductive span	5314	527/76/297	0.97 (0.95–0.99)	0.020	0.93 (0.89–0.97)	<0.001	0.98 (0.96–1.01)	0.251
Natural menopause age(years)	4580	446/56/245	0.97 (0.94–0.999)	0.043	0.94 (0.88–1.01)	0.110	0.98 (0.95–1.00)	0.309
Hysterectomy and/or oophorectomy <55 years								
yes	707	77/20/49	1.22(0.90–1.67)	0.197	2.60(1.48–4.58)	0.001	1.38(0.96–1-98)	0.078
no	4607	450/56/248	ref	ref	ref	ref	ref	ref

AD, Alzheimer’s disease, VaD Vascular dementia; Model 1 was adjusted for birth year. Model 2 was adjusted for birth year, education level, smoking, ApoE4 allele, number of children, diabetes, body mass index, alcohol use, physical inactivity

### Depression as a potential mediator between older menopause age and risk of dementia

The association remained after excluding persons with depression and the association was not significantly attenuated after adjusting for the presence of depression in HUNT2 (table 4).

**Table 4:** Assessing depression as a mediating factor in the association between menopause age and mild cognitive impairment/dementia in the HUNT4 70+ group

			**Model 1**		**Model 1**		**Model 2**		**Model 2**	
			**MCI**		**Dementia**		**MCI**		**Dementia**	
	**total No.**	**No. Cases MCI/ dementia**	**RR ratio** **(95% CI)**	***p*** **value**	**RR ratio** **(95% CI)**	***p*** **value**	**RR ratio (95% CI)**	***p*** **value**	**RR ratio (95% CI)**	***p*** **value**
Menopause age (years)	5314	1747/900	0.98 (0.97–0.99)	0.029	0.96 (0.94–0.98)	<0.001	0.99 (0.97–1.00)	0.127	0.96 (0.95–0.98)	<0.001
Menopause age (years) adjusted for depression additionally	5314	1747/900	0.99 (0.97–1.00)	0.039	0.96 (0.94–0.98)	<0.001	0.99 (0.97–1.00)	0.141	0.97 (0.95–0.98)	<0.001
Menopause age (years) excluding persons with depression	4757	1545/776	0.99 (0.97–1.00)	0.169	0.97 (0.95–0.99)	0,002	0.99 (0.97–1.00)	0.409	0.97 (0.95–0.99)	0.009

Model 1 was adjusted for birth year. Model 2 was adjusted for birth year, education level, smoking, ApoE4 allele, number of children, diabetes, body mass index alcohol use, physical inactivity, when analysing menopause age, natural menopause age and reproductive span as risk factors. The presence of depression was defined by a HADS (Hospital anxiety and depression scale) score of ≥8

In the sensitivity analysis, the associations were studied using complete cases only, which demonstrated similar results to the imputed data (supplementary table 1). When the HUNT4 70+ female participants were analysed in two groups (<80 and 80 years or older) using MCI and dementia as endpoints, a significant association with dementia was only found in the younger age group of <80 years which may indicate that death acts as a competing risk (table 5).

**Table 5:** The relationship between menopause age and mild cognitive impairment/dementia in the HUNT4 70+ group: stratified by age at HUNT4

			**Model 1**		**Model 1**		**Model 2**		**Model 2**	
			**MCI**		**Dementia**		**MCI**		**Dementia**	
	**Total No.**	**No. Cases MCI/ dementia**	**RR ratio (95% CI)**	***p*** **value**	**RR ratio (95% CI)**	***p*** **value**	**RR ratio (95% CI)**	***p*** **value**	**RR ratio (95% CI)**	***p*** **value**
Menopause age (years) in those <80 years at HUNT4	3163	1079/215	0.98 (0.97–0.999)	0.039	0.94 (0.91–0.96)	<0.001	0.98 (0.97–1.00)	0.143	0.94 (0.91–0.96)	<0.001
Menopause age (years) in those ≥80 years at HUNT4	2151	668/685	0.99 (0.98–1.02)	0.958	0.99 (0.96.1,01)	0.279	0.99 (0.98–1.02)	0.621	0.98 (0.96.1,01)	0.131

Model 1 was adjusted for birth year. Model 2 was adjusted for birth year, education level, smoking, ApoE4 allele, number of children, diabetes, body mass index alcohol use, physical inactivity

## Discussion

In this study of 5314 women from a population-based study in Norway, overall, we found that older menopause age and longer reproductive span was associated with a lower risk of dementia. When dementia subtypes were analysed, older menopause age was associated with a lower risk of both VaD and AD. Participants who underwent oophorectomy and/or hysterectomy <55 years had a higher risk dementia compared to those who did not have these procedures. Older age of natural menopause was also associated with a lower risk of dementia.

We additionally studied MCI as an end point, but did not find an association between menopause age, menarche age and MCI. MCI has not been studied as a separate endpoint previously; previous studies examined cognitive impairment (which encompasses both MCI and dementia cases) as an endpoint (10, 25). MCI in this cohort constitutes an heterogenous group, includes both non-amnestic and amnestic MCI, and a third of MCI cases will not develop dementia (26). Therefore, it may not be surprising that there was no significant association between MCI and the reproductive factors explored here.

When assessing the risk of dementia subtypes, it appeared that older menopause age was associated with a lower risk of both AD and VaD with a greater reduction for VaD. However, the association was no longer significant when those with oophorectomy/hysterectomy <55 years were excluded. Moreover, those who had surgery <55 years demonstrated a higher risk of vascular dementia compared to those without surgery. These factors may indicate that surgically inducing menopause is an important factor driving an association between menopause age and vascular dementia. However, it should be acknowledged that the number of participants who experienced vascular dementia was relatively low (n=76) and further studies with a larger number of participants with vascular dementia are warranted to examine this association more closely.

### Comparison to other studies

In our study, we found that reproductive factors linked to longer lifetime oestradiol exposure, such as experiencing natural menopause at an older age and having a longer reproductive span, were linked to a reduced risk of dementia. On the other hand, undergoing hysterectomy or oophorectomy before the age of 55, indicative of shorter oestrogen exposure, was associated with an elevated risk of dementia. Our study did not demonstrate an association between menarche age and dementia. A meta-analysis revealed that risk pattern between menarche age and all cause dementia was not significant which is similar to the result demonstrated here our study RRR 0.99 (95% CI 0.92–1.05) vs. metanalysis pooled RR=0.93 (95% CI 0.87 –1.00) (27). Menarche age is subject to less variation, as demonstrated here where 70% of women in our cohort experienced menarche between 12–14 years. Since menarche age is subjected to less variation (SD 1.4 years), it has less effect on the total reproductive span compared to menopause age (SD 4.6 years), and menarche age may therefore not have the same impact on lifetime oestrogen exposure. Gong et al (11) found an association between menarche age and dementia however the study population consisted of women with a median age of 55 years and a median follow up time of 10 years, thus demonstrating findings that would be applicable to those with younger onset dementia, and this corresponds to the smaller proportion of dementia cases found in their study (0.3% compared to 16.9% in our study). Our findings replicate findings on menopause age from most previous studies and a meta-analysis in 2016 (10, 11, 23, 28) whereas the Gothenburg study showed that older age of menopause was associated with higher dementia risk. Although both our study and the Gothenburg study were performed on Scandinavian populations, the studies differed in several aspects. The sample size was 1364 in their study whereas we had nearly 4 times as many participants (n=5314) and therefore likely greater power. This aspect likely had the most effect on the results. The regression analysis used in the study by Najar et al was Cox regression since time to event data were available with several timepoints of dementia status assessment. Multinomial logistic regression was used here, as dementia was assessed only at HUNT4. The results stratified by age of onset of dementia in the Najar et al study showed that the association was only present among those with dementia onset after age 75 years. We did not perform a similar analysis in this study due to lack of information on dementia onset. The covariates used for adjusting in their analysis were different to ours notably they adjusted for oestrogen related factors such as number of pregnancies, months of breastfeeding, exogenous oestrogen whereas we adjusted for number of children and other confounders in this study (8).

While this study and others have found that older menopause age is linked to a lower risk of dementia, observational studies on postmenopausal hormone replacement therapy (HRT) have been conflicting showing both increased and decreased risk of dementia (29, 30). These divergent results with HRT may reflect that the positive effect of older menopause age is not linked merely to hormonal aspects of menopause. Instead, it maybe that this relationship is more complex than previously thought, where other biological or environmental factors connected to menopause are driving this association. Depression was however not found to be a potential mediating factor in this study.

### Mechanisms

Age specific prevalence of dementia is higher in women than in men and cannot be merely ascribed to the increased longevity of women compared to men. Factors related to the female reproductive system and the reproductive period are unique to women and therefore it is important to assess their role in dementia risk. The effects of the reproductive period are often thought to be related to the lifetime exposure of oestrogen. The findings here support the hypothesis that longer exposure to oestrogen during the female lifespan as would be expected with longer menopause or reproductive span may have a protective effect against dementia. Many different protective mechanisms have been postulated. Oestrogen may be an important protective factor shielding neuronal mitochondria from amyloid-β toxicity (31). Oestrogen is postulated to increase neuronal connections in the hippocampal area and significantly reduced hippocampal volume in post-menopausal women compared to pre-menopausal women has been observed (32, 33). Premenopausal women have lower cardiovascular risk compared to age matched men and this reduced vascular risk could also confer benefits in terms of dementia risk (34).

### Strengths and limitations of this study

This study has several advantages. We present findings from a large population-based study of 5314 women. To minimise recall bias, majority of the data about menopause age were obtained from the HUNT2 study, closer to the event. However, we did not analyse inconsistencies in reporting of menarche age and menopause age between the HUNT waves. The diagnosis of MCI and dementia was classified using standardised methodology during the 2017–2019 HUNT4 70+ study and included cognitive assessments of participants who were nursing home residents. Using standardised methods to ascertain cognitive impairment and dementia throughout a population ensures a robust and consistent diagnostic process compared to using health records alone in previous studies (9, 11) which has been noted to result in low sensitivity (71%) for identifying all-cause dementia (35). We used both MCI and dementia as endpoints, encompassing a wider spectrum of cognitive impairment. We were also able to perform an exploratory analysis on dementia subtypes which demonstrated that older menopause age was significantly associated with a lower risk of both VaD and AD, with risk reduction being more pronounced for VaD. The UK biobank study by Liao et al also demonstrated the strongest estimates for VaD (28). The vast amount of information available in the HUNT study enabled us to adjust for a range of covariates including ApoE4 status.

A limitation of this study is that cognitive assessments were not performed in the previous HUNT studies (HUNT1-3) and therefore lacks a timeline perspective of cognitive deterioration and dementia onset. Nevertheless, this cohort study examines cognitive status in all women aged 70 years and older during the 2017–2019 HUNT4 70+ study where the individuals receiving a dementia diagnosis had a wide age range between 71 and 104 years of age. Given the life expectancy of 8–10 years after a dementia diagnosis this likely allows for a broad view of risk of all cause dementia at varying ages of onset likely ranging from before 70 years to over 85 years. This aspect allows for our findings to be more applicable to a general population with dementia of varied aetiology and different ages of onset. There may be some individuals in the original HUNT1 population who are already deceased due to dementia and therefore are not captured in this HUNT4 70+ sample. In addition, when stratified by age, dementia risk was evident only in the younger age group <80 years indicating that death may act as a competing risk possibly attenuating the magnitude of the association. Data on exogenous oestrogen use such as hormonal contraception and hormone replacement therapy were not included in this analysis. All reproductive data here is self-reported. Furthermore, the study participants consisted mainly of white European women which limits generalisation of the findings to other ethnic groups. Although adjustments were made for multiple covariates, there may still be other unmeasured factors that can lead to residual confounding.

## Conclusions

This study showing that older menopause age is associated with decreased risk of dementia demonstrates the importance of studying factors specific to women and the risk of dementia. It is important to try and ascertain if the risk reducing effects of increased menopause age are in fact linked to oestrogen exposure through further studies investigating endogenous oestrogen levels. Since hormonal contraception is widely used, it is also necessary to examine how the association between reproductive span and dementia is affected by using exogenous oestrogen during the reproductive period. If lifetime oestrogen exposure is important in dementia prevention, future work should aim to elucidate if the risk is due to direct oestrogen related effects on the brain or due to mediating factors such as the increased vascular risk after menopause or other comorbidities after menopause. Thus, future studies should explore which factors mediate or offset the association between menopause age and dementia and if post-menopausal hormone replacement therapy or other therapeutic interventions could modify this relationship.

## Electronic supplementary material

Supplementary material, approximately 36 KB.

## Notes

*Ethical standards:* All studies were conducted in line with the Helsinki Declaration. The Regional Ehtics Committee for Medical and Health Research approved this study (reference number 198809).
