# Supplementary material for: Female Reproductive Factors and Risk of Mild Cognitive Impairment and Dementia: The HUNT Study
Source: J Prev Alzheimers Dis. 2024 Mar 5;11(4):1063–72. doi: 10.14283/jpad.2024.46 (PMC11937202; doi:10.14283/jpad.2024.46)
Supplement: Supplementary file 1 — Supplementary material, approximately 36 KB. [file mmc1.docx]

**Supplementary table 1: The relationship between reproductive risk factors and mild cognitive impairment/dementia in the HUNT4 70+ group (complete cases only).**

|  |  | **Model 1** |  | **Model 1** |  | **Model 2** |  | **Model 2** |  |
| --- | --- | --- | --- | --- | --- | --- | --- | --- | --- |
|  |  | **MCI** |  | **Dementia** |  | **MCI** |  | **Dementia** |  |
|  |  | **RR ratio (95% CI)** | ***p value*** | ***RR ratio (95% CI)*** | ***p value*** | **RR ratio (95% CI)** | ***p value*** | **RR ratio (95% CI)** | ***p value*** |
| Menarche age (years) ^*^ | | 0.98 [0.93- 1.02] | 0.373 | 0.99 [0.92- 1.05] | 0.749 | 0.97(0.92-1.01) | 0.230 | 0.97(0.90-1.04) | 0.442 |
| Menopause age (years) ^*^ | | 0.99 [0.97- 1.00] | 0.027 | 0.97 [0.94- 0.99] | <0.001 | 0.99 (0.97-1.00) | 0.062 | 0.97[0.94- 0.99] | 0.001 |
| Natural menopause (years) ^*, †^ | | 0.98(0.96-0.99) | 0.009 | 0.95(0.93-0.98) | 0.001 | 0.98(0.95-1.00) | 0.136 | 0.96(0.94-0.99) | 0.037 |
| Reproductive span (years) ^*^ | | 0.99[0.98- 1.00] | 0.050 | 0.96 [0.95- 0.98] | <0.001 | 0.99 (0.98-1.00) | 0.164 | 0.97(0.95-0.99) | 0.005 |

Model 1 was adjusted for birth year. Model 2 was adjusted for birth year, education level, smoking, ApoE4 allele, number of children, diabetes, body mass index alcohol use, physical inactivity, when analysing menopause age, natural menopause age and reproductive span as risk factors. Model 2 was adjusted for age, education level, smoking, diabetes, alcohol use, physical inactivity, body mass index, when studying menarche age as a risk factor.

^*^as a continuous variable

^†^natural menopause excluding women who reported hysterectomy and/or oophorectomy <55 years.

No of cases

Menarche age model1 n=5186 model2 n=4314

Menopause age model1 n=5152 model2 n=4257

Natural menopause age model1 n=3755 n=3262

Reproductive span model1 n=5116 model2 n=4243
